# Effects of an instructional WhatsApp group on self-care and HbA1c among female patients with Type 2 diabetes mellitus

**DOI:** 10.1371/journal.pone.0305845

**Published:** 2024-09-18

**Authors:** Riham Saud Alhazmy, Asmaa Hamdi Khalil, Hayfa Almutary

**Affiliations:** 1 Faculty of Nursing, Medical/Surgical Department, King Abdulaziz University, Jeddah, Saudi Arabia; 2 Medical Department Rabigh General Hospital, Rabigh, Saudi Arabia; 3 Faculty of Nursing, Ain Shams University, Cairo, Egypt; BP Koirala Institute of Health Sciences, NEPAL

## Abstract

**Aims and objectives:**

To assess the effect of an instructional WhatsApp group on self-care and HbA1c levels among female patients with type 2 diabetes mellitus (T2DM).

**Background:**

T2DM is a chronic disease that requires effective self-care. WhatsApp is a free application that can be effectively used for patient education.

**Design:**

This study used a quasi-experimental design.

**Methods:**

A convenience sample of 62 female participants was recruited from the medical outpatient clinic of a tertiary hospital. The Diabetes Self-Care Scale was used to assess the self-care profiles of the participants pre- and post-intervention. HbA1c samples were also collected at baseline and three months after receiving instructions from the WhatsApp group. Sociodemographic and clinical data were collected during the pre-intervention stage.

**Results:**

The mean HbA1c level decreased from 8.61 ± 1.70 to 7.92 ± 1.60 after implementing the WhatsApp group instructions; the values showed a significant difference (t-value = 5.107 and *P*-value < 0.001). The post-test mean score of total self-care was higher than the pre-test mean score (t-value = 12.359, *P*-value <0.001), indicating a highly significant difference.

**Conclusions:**

The study demonstrated that the instructional WhatsApp group is an effective method for improving self-care and HbA1c levels in patients with T2DM. This study suggests the use of WhatsApp group instructions as a teaching method in the healthcare system for the education and follow-up of patients with T2DM.

**Relevance to clinical practice:**

The findings support the need to initiate effective and dynamic interventional follow-ups through WhatsApp groups for patients with T2DM to improve their self-care and HbA1c levels and ultimately reduce the burden on hospitals and governments.

## Introduction

Diabetes mellitus (DM) is an alarming global health issue that can lead to serious complications if uncontrolled or not managed appropriately. It is one of the fastest growing global health emergencies of the 21st century [1]. In 2019, DM reached pandemic proportions with a worldwide prevalence of 9% (463 million adults) [2]. More than half a billion people worldwide have developed DM, and approximately 1 in 10 adults have the disease [1]. The number of cases has increased over the past two years [1]. In addition, DM is one of the most common diseases that cause mortality and morbidity in Saudi Arabia. According to a review of national data, DM affects 8.5% of the total adult population of Saudi Arabia [3].

DM complications are associated with frequent and prolonged hospitalizations, which increase the burden on individuals and the healthcare system [4]. According to the American Diabetes Association (2018), the total estimated cost of a diabetes diagnosis in the United States in 2017 was $327 billion; this value includes medical costs and reduced patient productivity [5]. The chronic nature of this disease requires self-care and self-management to prevent possible complications.

The digital health revolution provides helpful tools that support healthy practices among people with chronic noncommunicable diseases (NCDs), such as diabetes [6]. This includes using mobile health applications in patients’ education. A growing number of studies demonstrate the effectiveness of using mobile apps for lifestyle changes and self-management in people with chronic NCDs such as diabetes, hypertension, and cardiac diseases [7,8]. According to a cross-sectional study with 1119 participants, the majority of respondents believed that using mobile health to prevent NCDs would be beneficial (62%), and that it would enable patients to manage their lifestyle modifications (59%) [9]. In addition, mobile apps were found to be effective tools for those in rural areas [8] and across all age groups [9]. However, choosing the appropriate mobile apps, such as WhatsApp, to enhance health still needs more investigation.

## Background

Type 2 diabetes mellitus (T2DM) is a metabolic disorder that occurs as a result of insulin resistance and impaired insulin production by islet β cells in the pancreas; this condition leads to elevated blood glucose levels, resulting in increased glycated hemoglobin (HbA1c) levels [10,11]. In 1990, T2DM was the 18th leading cause of mortality and the 9th cause of morbidity. In 2020, it ranked as the 9th cause of worldwide mortality and the 7th cause of morbidity [12]. According to the World Health Organization (2020), DM is the 7th leading cause of death among women in Saudi Arabia [13]. Various factors contribute to the increase in the total number of patients with DM, and they include an aging population and a rising obesity rate [14,15]. In addition, the rate of obesity in women is higher than that in men [16].

HbA1c is a diagnostic tool and objective measure that healthcare providers and researchers use to assess the clinical outcomes of patients with diabetes. It represents the average blood glucose levels of individuals over the previous 2–3 months based on the presumed half-life of red blood cells [17]. According to the Saudi Diabetes Clinical Practice Guidelines, the normal HbA1c level is 4%–5.6% [18]. The American Diabetes Association (2021) recommended that A1c should be less than 7.0% in adults with diabetes [19], and A1c 8% indicates poor diabetes control [17].

DM is a complex, long-term illness that requires regular medical assistance and multifaceted risk-reduction methods that are beyond glucose management. Given the unavailability of a definitive cure, secondary prevention is the best approach. Appropriate patient education regarding self-care can delay or prevent the onset of acute and chronic complications [20]. Self-care involves many aspects, such as diet, physical activities, medication adherence, blood glucose monitoring, problem solving, and coping skills [21]. The critical element for controlling diabetes is patients’ self-care management.

Several recent studies suggested the use of diabetes self-management education (DSME) to control the disease [22–24]. In these studies, the HbA1c levels of patients with T2DM who participated in DSME decreased by 0.71%–1.57% relative to those of patients on standard therapy [22–24]. With the development of technology, mobile applications have been broadly used to communicate and deliver information in a simple and easy manner. WhatsApp is a free messenger application that can be used across multiple platforms such as Android and iPhone devices [25]. Instructional WhatsApp groups can create a competitive environment to decrease the level of glycosylated hemoglobin by providing instructions by educators. Additionally, group members can encourage one another to achieve their primary goals [26]. The recent literature suggests that WhatsApp is an effective medical learning tool [27]. In Saudi Arabia, 71% of the total population uses WhatsApp, and people spend an average of three hours and two minutes on social media [25]. In addition, a study conducted in Saudi Arabia showed that Saudi women tend to learn through WhatsApp [28]. The theoretical framework for this study is based on the trans-theoretical model (TTM) of stages of change established by James Prochaska and Carlo DiClemente (the 1980s) [29]. The model has been used to help people develop healthy behaviors, including weight loss, exercise, and quitting unhealthy behaviors. In addition, it presents a health-promotion strategy that considers behavioral change as a series of steps [30]. At present, few studies have focused on the effects of instructional WhatsApp groups on self-care and HbA1c levels in female patients with T2DM, especially those in Saudi Arabia. Hence, the current study aimed to assess the effect of instructional WhatsApp groups on self-care and HbA1c levels among female patients with T2DM. Therefore, the findings of this study may help identify new strategies for managing T2DM.

### Research objectives

The aim was achieved through the following objectives:

1. Assessing the level of self-care and HbA1c among type 2 diabetic female patients.
2. Providing diabetes self-care-related instruction through WhatsApp group.
3. Measuring the effect of instructional WhatsApp group on self-care and level of HbA1c among type 2 diabetic patients.

### Research hypothesis

• Type 2 diabetes female patients’ self-care will improve post implementing WhatsApp group instruction.

• HbA1c will decrease among female patients with type 2 diabetes post implementing WhatsApp group instruction.

## Materials and methods

### Design

A quasi-experimental design (pre- and post-test) was used in this study.

### Participants

The inclusion criteria were female adults with T2DM who could read and write Arabic and use WhatsApp on their cellphones. The exclusion criteria were patients using a different application, pregnant women with gestational diabetes, post-operative patients, patients with hearing or visual disabilities, and those with other comorbidities that would prevent them from participating in the study (e.g., mental illness and cerebrovascular accident).

**Fig 1:**
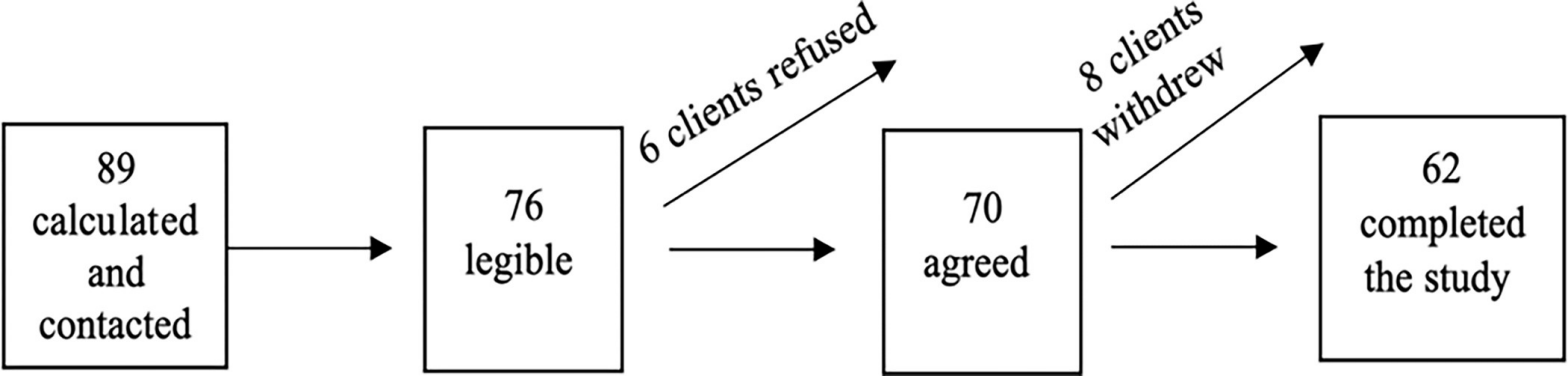
Particepant recruitment flowchart.

Data were gathered from the medical outpatient clinic of the Rabigh General Hospital in the Western region of Saudi Arabia. The sample size was 89, which was calculated using the Raosoft program based on a report of the population size at this hospital in 2020 (114 female patients with T2DM), a level of confidence of 95%, a margin of error of 0.05, and a probability value of 0.5. A total of 70 patients who met the inclusion and exclusion criteria were recruited to participate in the study; 8 of them withdrew from the study during the intervention phase (3 left the WhatsApp group, and 5 did not complete the post-test). Therefore, the final sample size was 62 patients who completed three months of the WhatsApp intervention and the post-test (Fig 1).

### Data collection

The data collection process was divided into three phases: pre-test, intervention, and post-test.

#### Pre-test phase

After obtaining ethical approval, the researcher met with the head nurse of the clinic to facilitate data collection. Initially, the medical records of female patients with diabetes were reviewed to identify those who met the inclusion criteria. Patient names, file numbers, and phone numbers were recorded to facilitate the communication with potential participants (files without phone numbers were excluded).

Female patients with T2DM were contacted to check if they could read and write Arabic and if they had a smartphone with the WhatsApp application. A total of 76 females who met the inclusion criteria were invited to participate in the study; 6 of them refused to participate. Those who agreed to participate in the study signed an informed consent form and were then provided with pre-test questionnaires to gather baseline data about their self-care. Blood samples were collected for HbA1c analysis.

#### Intervention phase

The duration of the intervention phase was three months. Initially, the WhatsApp group was created and moderated by one researcher who is a registered nurse and diabetes educator and two other researchers who are associate professors in medical–surgical nursing. Instructions regarding diabetes self-care were provided through the WhatsApp group in the form of pictures, videos, and daily messages. The WhatsApp group was open daily from Sundays to Thursdays, from 6:00 p.m. to 8:00 p.m., for discussions, questions, or any clarifications or concerns. All instructions were sent to the group daily, and questions were answered by the moderator accordingly. Private conversations were not permitted. Depending on the amount of information presented during the first month, each topic was presented for 1–3 days. Patients were motivated to follow the instructions in the WhatsApp groups for the next 2 months.

#### Post-test phase

After the completion of the intervention phase, an appointment schedule was sent for the post-test and HbA1c analysis and was sent to the patients. The patients were divided according to their code numbers, with 15–19 patients scheduled for the post-test and HbA1c analysis per day. The patients were invited again the day before the appointment, and a private message through WhatsApp was sent on the day of the appointment as a reminder. After completing the same questionnaires, a blood sample was withdrawn for HbA1c analysis. The results were then compared with the previous ones.

### Measures

Data were collected using two structured, validated questionnaires. The first one was aimed at assessing the patients’ demographic and clinical data. It included demographic data such as age, marital status, level of education, and working status. It also covered clinical data such as duration of disease, methods used to treat diabetes, family history related to diabetes, comorbidities, and education about self-care for diabetes. The second questionnaire used was the Diabetes Self-Care Scale. This scale was adapted from Lee and Fisher (2005) and modified in the current study to measure self-care practices related to diabetes [31]. The modified scale includes 28 statements that are related to self-care activities and are grouped into seven domains. These domains are dietary control (five statements); exercise (three statements); blood glucose monitoring (two statements); medication adherence (three statements); follow-up (three statements); foot care (five statements); and other self-care practices related to hygiene, diabetes identification, and avoidance of complications (seven statements). The responses to these statements were rated on a 6-point Likert scale, with the choices ranging from 1 “strongly disagree” to 6 (“strongly agree”). The scores for each domain and the total scale were calculated, and the mean scores were calculated and categorized as good, moderate, or poor. The intervals between the three categories were calculated by subtracting the lowest value from the highest value for every domain and the total and then dividing the results by 3.

### Ethical consideration

Ethical approval was obtained from the Ethics Committee of the Faculty of Nursing, King Abdulaziz University (Ref No. 2M. 79) and from the National Board Review of the Ministry of Health at Jeddah Research Center (IRB No. H-02-J-002) to collect data from the hospital in the Rabigh, Makkah region where the study was conducted.

Written informed consent was obtained from all participants who agreed to participate in the study after explaining the research objective during the interviews. A summary of the search, purpose, duration, advantages, and disadvantages of the intervention was provided in Arabic. The ethical aspects of the study were based on research ethics and principles. The patients were informed that their participation was voluntary and that they had the right to continue or withdraw. Confidentiality and anonymity were protected by providing a code number for each participant at the data collection stage. In addition, all data gathered during the study were kept confidential, and only the researchers had access to personal information.

### Statistical analysis

Data analysis was performed using the Statistical Package for Social Science (version 23.0). Descriptive analyses using frequency, percentage, mean, and standard deviation (SD) were performed to determine the distribution of the study participants’ sociodemographic variables and clinical data. Normal distribution was evaluated using the kurtosis and skewness test and Jarque–Bera test. A paired sample t-test was used to compare the self-care domains and HbA1c before and after implementing the WhatsApp group instructions. The significance of the results was categorized using P-values: *P* ≤ 0.05 was considered statistically significant; *P* ≤ 0.01, *P* ≤ 0.001 was considered highly statistically significant, and *P* > 0.05 was considered non-significant. Cohen’s d was used to measure the effect size, with d ≤ 0.2, 0.2 < d < 0.8, and d > 0.8 indicating small, moderate, and large effect sizes, respectively.

## Results

### Demographic and clinical characteristics

Table 1 presents the participants’ demographic characteristics. The mean age of the study participants was 47.6 ± 9.74. Most study participants were married (66.1%) while a few (8.1%) were single. Regarding education level, 58.1% of the study participants had less than a secondary level of education while 17.7% had a bachelor’s degree. In terms of occupation, 77.4% of the study participants were not employed.

**Table 1:** Demographic and clinical characteristics of study of participants (n = 62).

Demographic Characteristics		No.	%
**Age (Mean± SD)**	47.6±9.74		
	25–35 yrs.	11	17.7
	36–46 yrs.	16	25.8
	47–57 yrs.	26	42
	≥68 yrs.	9	14.5
**Marital status**	Single	5	8.1
	Married	41	66.1
	Divorced	8	12.9
	Widowed	8	12.9
**Educational level**	Less than secondary	36	58.1
	Secondary school	15	24.2
	University and above	11	17.7
**Occupation**	Not working	48	77.4
	Retired	4	6.5
	Worker	10	16.1

*Note*: SD = standard deviation.

The clinical characteristics of the patients are presented in Table 2. Approximately one-quarter of the sample (21%) had T2DM for 15 years or more, and only a small percentage (8%) had T2DM for less than a year. In terms of treatment, 58.1% of the participants used oral antidiabetics while 4.8% used diet and exercise. Furthermore, 64.5% of the patients had a family history of diabetes. More than half of them (54.8%) indicated that they had previously received diabetes self-care education.

**Table 2:** Clinical characteristics of the study participants (n = 62).

Clinical Characteristics	Category	No.	%
**Duration of diabetes**	< 1 year	5	8.1
	1 - < 5 years	12	19.3
	5 - < 10 years	16	25.8
	10 - < 15 years	16	25.8
	≥ 15 years	13	21
**Diabetic treatment type**	Diet and exercise	3	4.8
	Oral antidiabetics	36	58.1
	Insulin	8	12.9
	Oral antidiabetics and insulin.	10	16.1
	Oral antidiabetics before and insulin now	5	8.1
**Family history of diabetes**	Yes	40	64.5
	No	22	35.5
**Other comorbidities**	Yes	19	30.6
	No	43	69.4
**Receiving diabetic self-care education**	Yes	34	54.8
	No	28	45.2

### Diabetes self-care among study participants before and after the implementation of the WhatsApp group instructions

As shown in Table 3, the paired samples t-test was used to compare the mean scores of the self-care domains among the study participants before and after implementing the WhatsApp group instructions at a significance level of α = 0.05. Moreover, the effect size was calculated using Cohen’s d, with the values d ≤ 0.2, 0.2 < d < 0.8, and d ≥ 0.8 indicating small, moderate, and large effect sizes, respectively.

**Table 3:** Mean Scores of self-care domains among study participants pre and post implementing WhatsApp group instructions.

Self-Care domains	Ranges	Pre	Post	Paired-sample T test	P value Sig.	Cohen’s d
		M ± SD	M ± SD			
**Dietary control**	5–30	16.84 ± 6.63	21.06 ± 5.90	5.176	<0.0001*	0.657
**Exercise**	3–18	6.79 ± 4.57	12.68 ± 3.84	10.079	<0.0001*	1.280
**Blood glucose monitoring**	2–12	4.53 ± 2.88	8.85 ± 2.88	10.479	<0.0001*	1.331
**Medication adherence**	3–18	11.76 ± 4.46	14.15 ± 3.48	4.237	<0.0001*	0.538
**Follow up**	3–18	11.48 ± 4.93	16.02 ± 2.77	6.478	<0.0001*	0.823
**Foot care**	5–30	20.08 ± 4.93	24.79 ± 4.98	6.725	<0.0001*	0.854
**Others**	7–42	24.87 ± 5.79	34.55 ± 6.12	10.921	<0.0001*	1.387
**Total**	28–168	96.35±21.39	132.10±22.49	12.359	<0.0001*	1.570

*Note*: SD = standard deviation; M = mean; * p < 0.001 high significant.

With regard to dietary control, Table 3 shows that the post-test mean score is higher than the pre-test mean score with a calculated t value = 5.176 and P-value < 0.001, indicating a highly statistically significant difference between them. Measuring the effect size of the implementation of the WhatsApp group instructions on the level of dietary control revealed a Cohen’s d = 0.657 (> 0.2 and < 0.8), indicating that the effect of the implementation was moderate.

Regarding the exercise domain, the Table 3 shows that the post-test mean score was higher than the pre-test mean score. The calculated paired t-value = 10.079 and P-value < 0.001 denoted the highly statistically significant difference between the scores. Specifically, the level of exercise among the study participants increased and improved because of the implementation of the WhatsApp group instructions, with Cohen’s d = 1.280 > 0.8, which indicated a large effect size.

With regard to blood glucose monitoring, the same table shows that the post-test mean score was higher than the pre-test mean score. The calculated paired t-value was 10.479 while the P—value < 0.001, indicating the highly statistically significant difference between the scores. Measuring the effect size of the implementation of the WhatsApp group instructions on the level of blood glucose monitoring revealed a Cohen’s d = 1.331 > 0.8, indicating a large effect.

Regarding medication adherence as a self-care domain, Table 3 shows that the post-test mean score was higher than the pre-test mean score. The paired t-value = 4.237 and P-value < 0.001 indicated a highly statistically significant difference. Measuring the effect size of the implementation of the WhatsApp group instructions on the level of medication adherence based on Cohen’s d revealed a value of d = 0.538 > 0.2, indicating a moderate effect.

In relation to the participants’ follow-up before and after implementing the WhatsApp group instructions, the same table shows that the post-test mean score increased more than the pre-test mean score, with the tabulated t-value being 6.478 and P-value < 0.001, which indicated the highly statistically significant difference between them. Measuring the effect size of the implementation of the WhatsApp group instructions on the level of follow-up using Cohen’s revealed a value of d = 0.823 > 0.8, which indicated a large effect.

Regarding foot care after implementing the WhatsApp group instructions, Table 3 shows that the post-test mean score was higher than the pre-test mean score. The paired t-value = 6.725 and P-value < 0.001 indicated the highly statistically significant difference between them. The effect size of implementing the WhatsApp group instructions was large, with Cohen’s d = 0.854 > 0.8.

For the other self-care practices related to hygiene, diabetes identification, and avoidance of complications, Table 3 shows that the post-test mean score was higher than the pre-test mean score. The paired t-value was 10.921 while the P-value < 0.001, indicating a highly statistically significant difference. The effect size of implementing the WhatsApp group instructions was large, with Cohen’s d = 1.387 > 0.8.

In relation to total self-care, the post-test mean score was higher than the pre-test mean score. The t-value was 12.359 while P-value < 0.001, indicating a highly statistically significant difference between them. The effect size of implementing the WhatsApp group instructions on the level of total self-care was large, with Cohen’s d = 1.570 > 0.8.

### HbA1c among study participants before and after the implementation of the WhatsApp group instructions

Table 4 illustrates the difference between the mean score of the HbA1c levels among the study participants before and after the implementation of the WhatsApp group instructions. The mean HbA1c level among the study participants decreased from 8.61 ± 1.70 to 7.92 ± 1.60 after implementing the WhatsApp group instructions. The t-value = 5.107 and P-value < 0.001, with an absolute reduction of 0.69, indicated the highly statistically significant difference between them. The effect size of implementing the WhatsApp group instructions on the level of HbA1c was moderate given Cohen’s d = 0.649 > 0.2.

**Table 4:** Mean score of Hba1c among study participants pre and post implementing WhatsApp group instructions.

	Normal range of HbA1c	Pre-test Mean ± SD	Post-test Mean ± SD	T-test	P-value	Cohen’s d
**HbA1c level**	4–5.6	8.61 ± 1.70	7.92 ± 1.60	5.107	<0.001*	0.649

*Note*: * p < 0.001 high significant.

## Discussion

The current study demonstrated the effectiveness of using the instructional WhatsApp group on self-care and HbA1c among female patients with T2DM. Subjective and objective data were used to assess the changes in clinical outcomes. The results showed that the instructional WhatsApp group improved all domains of self-care and HbA1c levels.

Regarding dietary control, the mean score of all dietary control items increased, indicating an improvement in self-care under this domain. This finding is in line with those of previous studies [22,32]. Changing one’s lifestyle, particularly eating habits, necessitates regular reminders and increased motivation. In this regard, WhatsApp group instructions appear to be an effective method.

The study showed that the total mean scores in the exercise domain improved significantly after the implementation of the WhatsApp group instructions. This finding is consistent with the study of ElGerges (2020), who used traditional education and followed the patients for three months; their study revealed a significant improvement in the exercise domain [22]. A similar finding was reported in another study conducted in Saudi Arabia that measured the effect of a WhatsApp-based intervention on promoting physical activity among female college students in Abha [33]. This study found that social network-based interventions (WhatsApp) contribute to improvements in physical activity. However, some studies revealed that the exercise domain did not improve significantly after participation in the studies [32,34,35]. These findings could be due to the continuous support and encouragement through the group in which participants are asked to download a step-counting application and share photos of their walking areas after receiving the related knowledge.

For blood glucose monitoring, the total mean scores improved significantly after the implementation of the WhatsApp group instructions. These findings are congruent with those of ElGerges (2020) and Zheng et al. (2019), who reported a positive relationship between DSME and blood glucose monitoring and a significant improvement in the post-test mean scores in relation to blood glucose monitoring [22,36]. Meanwhile, Dinar et al. (2019) and Hailu et al. (2019) found no relationship between DSME and blood glucose monitoring [32,34]. The discrepancy in some of the findings across studies may be related to several factors, including the strategies used to remind participants. The findings of the current study may be attributed to the fact that the patients were constantly reminded of the need to monitor their blood glucose, document it, and analyze the readings. In addition, notebooks were distributed to the participants during the pre-test visit to record their blood glucose levels. These practices motivated the participants to follow instructions and change their lifestyle.

Medication adherence in patients with chronic diseases remains challenging. The clinical outcomes of patients with DM are usually related to medication adherence. In this study, an instructional WhatsApp group was used to assess its effect on patient adherence to medications. The results showed that the total mean score of the medication adherence domain improved significantly after the implementation of the WhatsApp group instructions. This finding is consistent with those of ElGerges (2020) and Zheng et al. (2019), who found positive patient outcomes regarding medication adherence after implementing diabetes self-management education [22,36]. Furthermore, a study conducted in the Kingdom of Saudi Arabia (KSA) showed that compliance rates for individuals with diabetes range from 60% to 80% for insulin and from 65% to 85% for oral antidiabetic drugs [37]. However, Sartori et al. (2020), who used the WhatsApp application to assess the impact of education on medication adherence, reported that the findings were clinically significant but not statistically significant [38]. In addition, previous studies revealed that medication adherence for T2DM did not improve significantly with the WhatsApp application [34,35]. Regardless of these differences in the findings of previous studies, the recent literature has reported high medication compliance among patients with diabetes after using WhatsApp group instructions [37]. Increasing knowledge, awareness, and correction of concepts related to medicines through WhatsApp groups may convince patients and contribute to great adherence to medicines.

This study also found that the intervention had a positive effect on patient follow-up. The total mean score in this domain improved significantly after the implementation of the WhatsApp group instructions; this result is similar to the findings of a previous study [34]. The use of WhatsApp instructions contributed to increased compliance with follow-ups through online clinical and face-to-face visits. Consultations with physicians when experiencing extremely high or extremely low blood glucose levels also increased. Not following-up is usually due to the fear of censure from healthcare providers and concerns about laboratory results. Such issues are often attributable to noncompliance with medical regimens. In the current study, the patients showed high compliance with their regimens. The motivation for patients to visit the clinic may be their enthusiasm for knowing their laboratory results after committing to medication, exercise, and nutrition.

The current study also demonstrated significant improvements in foot care following the implementation of the WhatsApp group instructions. Several studies have reported similar findings [22,32,34,36]. Patients with DM seem to be interested in this aspect. In addition, KSA is a country of Islam and Muslims who pray five times a day. Hence, the feet should be inspected five times as well through ablution (*wudu*). During the study intervention, the patients were encouraged to practice foot care by giving them simple instructions to follow and reminding them continuously. In addition, the complications associated with diabetic feet were explained to them.

For other self-care practices related to hygiene, diabetes identification, and avoidance of complications, significant improvements were noted in the mean scores following the implementation of the WhatsApp group instructions. In the WhatsApp group, the patients were encouraged to wear diabetes identification, maintain their self-cleaning regimen to prevent infections, and search the Internet or ask a healthcare provider when they have a new issue. In doing so, they may increase their awareness regarding these points.

Overall, self-care improved significantly following the implementation of the WhatsApp group instruction. This result is consistent with studies that lasted for three months [22,24,36]. By contrast, Waller et al. (2021) revealed that the total mean self-care score of the patients with T2DM did not improve significantly [35]. The positive findings regarding self-care in our study may be attributed to the fact that the instructions given through WhatsApp were carefully designed to suit different age groups according to their educational and social levels. The instructions were also validated by specialists in the field (endocrine consultant, medical consultant, and diabetic educator). Each part of the self-care program was provided separately, and feedback was obtained daily to ensure adherence to the recommended instructions. In the group, the patients were encouraged to share their experiences with one another through a group chat where they also shared new food recipes with pictures. Thus, they were motivated to learn and follow the instructions to achieve their goals.

The current study assessed the effect of the instructional WhatsApp group on HbA1c and found a significant improvement in the level of HbA1c with an absolute reduction of 0.69 after implementing the WhatsApp instructions. Previous studies also found a positive impact of using WhatsApp groups on HbA1c levels [22,23,26,39–41]. A few studies also demonstrated an improvement in HbA1c [35]. Often, commitments in the overall domains (i.e., diet control, exercise, blood glucose monitoring, medication adherence, foot care, and follow-up in self-care) would reflect objective results such as HbA1c results. In addition, sharing blood glucose level readings during daily follow-up could contribute to improving HbA1c levels.

The strength of this study lies in using a convenient teaching method through a WhatsApp group for giving instructions to female patients with type 2 DM and assessing its effect on their self-care and HbA1c level, where there is scanty research on this field. However, this study had some limitations. There was no follow-up measurement for self-care or HbA1c after six months or one year. Thus, longitudinal studies are recommended to assess the continued benefit of the intervention. Also, using a small sample size from one clinical site could restrict the generalizability of the study to Saudi Arabia. In addition, the quasi-experimental designs may be associated with the Hawthorne affect [42]. To reduce the possibility of this bias, we use an objective measure (the HbA1c test) to evaluate the effectiveness of the applied intervention.

## Conclusions

T2DM is one of the most remarkable diseases of the 21st century, threatening patients’ physical and psychological well-being. The instructional WhatsApp group effectively improved patient self-care and HbA1c levels. We recommend the adoption of WhatsApp group instructions as a teaching method in the healthcare system for the continuous education and follow-up of patients with diabetes.

### Relevance to clinical practice

Nurses in administration, bedside, and clinics play an important role in providing care to females with T2DM. Secondary prevention is required to avoid or prevent complications and involves the development of standardized guidelines to improve self-care practices and HbA1c levels among patients in healthcare centers and hospitals. These guidelines include the following: providing T2DM patients with a recording book before discharge; assessing self-care levels during hospital discharge and during the follow-up period in the clinics; and initiating effective, dynamic interventional follow-up through WhatsApp groups for female patients with T2DM to improve their self-care and HbA1c levels.

## Supporting information

S1 Dataset (SAV)
